# Developing an interplay among the psychological barriers for the adoption of industry 4.0 phenomenon

**DOI:** 10.1371/journal.pone.0255115

**Published:** 2021-08-02

**Authors:** Asif Mahmood, Asif Arshad Ali, Muhammad Nazam, Muhammad Nazim

**Affiliations:** 1 Department of Business Studies, Namal Institute, Mianwali, Pakistan; 2 Institute of Quality and Technology Management, University of the Punjab, Lahore, Pakistan; 3 Institute of Business Management Sciences, University of Agriculture Faisalabad, Faisalabad, Pakistan; 4 Department of Management Sciences, Khwaja Fareed University of Engineering and Information Technology, Rahim Yar Khan, Pakistan; Gonbad Kavous University, ISLAMIC REPUBLIC OF IRAN

## Abstract

This research aims to identify, rank, and create an interplay among the psychological barriers to adopting Industry 4.0 technologies in the manufacturing sector. A comprehensive literature review tracked by a discussion with industry and academic experts recognized 20 barriers. Based on three widely acclaimed statistical techniques, hybrid AHP-TOPSIS (Analytical Hierarchy Process-Technique for Order Performance by Similarity to Ideal Solution) and ISM (Interpretative Structural Modeling), critical psychological barriers have been investigated. A group of 8 experts from industry and academia with at least 10 years of experience was consulted for AHP and ISM techniques. Whereas TOPSIS was conducted by 443 operational-level users, including managers and supervisors of different functional areas of the manufacturing industry located in Pakistan. The findings reveal that ‘Fear of job losses’, ‘Fear of data loss/Risk of security breaches, ‘Lack of advanced & continued education of employees’ and ‘Lack of standards and reference architecture’, with highest importance weights, emerged as the most prominent psychological barriers in developing economies. Then the interrelations among these barriers resulted in a four-layered structural model. The driver barriers identified in the final model advocate that development in ‘advanced & continued education of employees’, ‘standards & reference architecture’ and ‘minimization of fear of job & data loss’ can expedite the adoption of industry 4.0 (i4.0) technologies. The study uniquely develops hierarchical relationships among the psychological barriers for adopting i4.0 in the manufacturing context using AHP-TOPSIS and ISM techniques. The study would be valuable for practitioners, decision-makers and companies that wish to focus their efforts and resources on removing the most critical barriers and challenges for the seamless implementation of Industry 4.0.

## 1. Introduction

The first industrial revolution, characterized by production facilities powered by steam engines, started at the end of the 18^th^ century. Then the second industrial revolution, marked by mass production through electricity and scientific management, took place at the beginning of the 20^th^ century. While, the third industrial revolution differentiated by automation, electronics and information technology began in the 1970s [1]. Now, the fourth imminent industrial revolution (aka industry 4.0) can be contemplated as new avenues of production such as the horizontal expansion of information and communication technologies (ICTs), learning machines, autonomous robots and complete digitization of the supply chain [2]. In fact, Industry 4.0 (i4.0) has opened new avenues of technological advancements and innovations, and is not the end of technological progress [3]. This fourth revolution can also be described as a set of smart factories, cloud computing, the internet of services, cyber-physical systems and the internet of things [4]. The concept of these smart factories surfaced in 2011 with the commencement of the German Government’s digital manufacturing project, a part of the erstwhile high-tech strategy at one of the largest trade fairs, ‘Hannover Messe’ [5]. I4.0 is not just a new manufacturing era [6] but has emerged as the most prominent solution achieving sustainability [7]. However, this paradigm shift from embedded to cyber-physical systems would put manufacturing organizations in a number of technological, organizational, and managerial challenges. Furthermore, the future production systems would require a set of new respective competencies because some processes are expected to be simplified, and others to become much more embedded and complex [8].

In this backdrop, organizations are more and more interested in searching for ways to be more agile to forthcoming changing patterns concerning product life cycles, variety, consumer expectations, and to stay ahead of the competition [9, 10]. However, being a novel area, this concept is still understudied [8]. Therefore, most organizations do not possess an organization-wide strategy to embrace it [11]. But this phenomenal technological thrust calls for investigation in all areas to understand and facilitate the transition. Furthermore, the revolution has various opportunities and barriers, but little literature is available concerning it [3]. Notwithstanding, some researchers have identified certain obstacles that may hold back manufacturers to attune to i4.0 [8, 12, 13]. For example, poor value-chain integration, economic benefits’ uncertainty, lack of infrastructure, job disruptions, resistance to change [14], cybersecurity issues, standardization problems, lack of skilled workforce [15, 16], expiration of existing business models [17] and organizational resistance at both employee and middle management level [8] may be particular issues/psychological barriers. Similarly, some factors positively affect i4.0 adoption, such as human resources, production systems-based resources, project management resources, management leadership-based resources, green logistics & design resources, information technology, big data analytics, and collaborative relationships [18]. However, psychological and behavioral barriers have not been appropriately addressed in the literature [19]. Several studies are limited to the technological side, insufficiently considering social aspects [20, 21]. Hence, there is a lack of studies in the latest literature that identify and create causal relationships among the psychological barriers to adopting the i4.0 phenomenon [22].

Hence, it is imperative to explore and comprehend the psychological barriers from organizational, management and technological perspectives because these aspects are still in their infancy [8]. These barriers are primarily caused by psychological conflicts owing to beliefs [23]. They are important also because managers’ perception of barriers, directly and indirectly, affects the actual application of industry 4.0 [24]. Thus, it reflects that more studies and evidence are desirable for a balanced decision because the domain is not mature enough. Based on these gaps, the following research questions arise: First, what critical psychological barriers might organizations encounter in implementing i4.0 technologies? Second, what is the priority order of these barriers? Third, what are the causal relationships among the shortlisted barriers? Fourth, what are the most prominent psychological obstacles possessing high driving power? In order to address these questions, the study has been designed to identify, rank, and then create causal links among the most critical psychological barriers of i4.0 implementation based upon experts’ opinions from industry and academia as well as managers and supervisors. Analytical Hierarchy Process (AHP) was applied to determine the relative weights of barriers, and the final ranking was obtained by conducting a Technique for Order Performance by Similarity to Ideal Solution (TOPSIS) analysis. Then interpretive structural modeling (ISM) has been employed to establish links among the shortlisted obstacles. Moreover, the psychological barriers have been classified into independent, dependent, linkage and autonomous categories through cross-impact matrix multiplication applied to classification (MICMAC), taking input from ISM. In this way, the most prominent psychological barriers (with high driving power) that hamper the i4.0 phenomenon have been extracted.

From a practical point of view, this research would be beneficial to the managers of manufacturing organizations in understanding not only the critical psychological barriers but also their interrelations to the adoption of the industrial revolution, and flooring the way for Industry 4.0’s successful implementation.

The rest of the paper has been organized as follows: Section 2 illustrates the most commonly reported psychological barriers of i4.0 based on the state of research presented in Section 1. Next, section 3 describes the research design, followed by the empirical results displayed in Section 4. Then, a detailed discussion has been carried out in section 5, in which sub-sections 5.1 and 5.2 highlight the theoretical contributions and managerial implications. Finally, section 6 draws conclusions, and section 7 elaborates limitations and future research directions.

## 2. Literature review

### 2.1. Article selection

Before initiating any research, exploring the existing work is essential [25]. But prior to conducting a literature review, consideration of the most relevant articles is necessary. Likewise, ensuring the quality and comprehensiveness of collected writings is also important. Therefore, a systematic literature review approach was utilized in the current study [7, 26, 27]. The review aimed to capture a snapshot of the diversity of research being conducted in the field of industry 4.0. An initial search of articles was conducted on Google Scholar, Web of Science and Scopus databases. The following keywords were used to search relevant articles: “industry 4.0”, “industry 4.0 adoption challenges”, “psychological barriers to adopt industry 4.0” and “driving and dependence power of industry 4.0 barriers”. The initial search retrieved 468 journal articles covering the journal articles published mostly between 2013 and 2021.

The journals included were from well-reputed publishers like Wiley Publication, Springerlink, Science Direct and Emerald. These databases provide full-text access to thousands of high-quality articles. There may be an article not surveyed in this paper, though the compiling efforts have been made to include the maximum number of articles.

The final shortlisted papers were individually studied to determine their relevance for the present research. Initially, industry 4.0 barriers addressed in these studies were mapped in an excel sheet, and later the repeating barriers were eliminated. The journal articles from management, computer science and engineering were included. Moreover, the journal articles were the focus of this search to ensure quality.

### 2.2. Barriers to the adoption of industry 4.0

A determining factor for industry 4.0 is the technological revolution [28]. The term “Industry 4.0” was coined very first at the Hanover Fair in 2011. It was adopted as a strategic initiative by the German Government for revolutionizing the manufacturing industry in 2013 [29]. Because of the various benefits of i4.0 to manufacturing organizations, it has gained increasing attention recently. But it is still in the embryonic phase regarding academic research [8]. The researchers have mainly conducted literature reviews in this area [30–32].

Concerning the implementation of Industry 4.0, Hofmann and Rüsch [31] argue that management faces challenges due to the unavailability of short-term financial returns. Furthermore, according to an analysis by Haddud, DeSouza [33], the integration of i4.0 into supply chains is associated with potential benefits and challenges. In developing economies, where cheap labor will no longer remain an added advantage, i4.0 poses various kinds of barriers [3]. However, the researchers agree that the investigation of barriers related to i4.0 implementation remains unexplored in the extant literature [8, 22, 32, 34]. Therefore, the discussion of such challenges or barriers to i4.0 will be presented here because understanding these barriers is important in implementing emerging and digital manufacturing technologies [35].

Kamble, Gunasekaran [22], Müller [36] and Müller, Kiel [13] all agree that one of the major challenges to implementing i4.0 is the fear of job losses. Either to fight or flight is the reaction of individuals to fear of the unknown. Employee acceptance is an essential factor for the success of Industry 4.0. Motivation and acceptance of problems are big obstacles to realizing the efficiency advantages and i4.0 introduction. One of the possible reasons for low employee acceptance is fear of job losses. There is a perception of inefficiency in employees regarding their competencies that leads to losses of jobs. Moreover, i4.0 demands IT-related competencies that traditional manufacturing job profiles do not cover [36]. This lack of skills threatens the adoption of i4.0 technologies [37]. Employees in developing nations face a hit where cheap labor is the key resource with technological advancements [20]. However, there is a dearth of understanding of Industry 4.0’s strategic significance [38], but such a fear can be reduced by providing continued education to the employees [39]. At the same time, the provision of advanced schooling for personnel training is another challenge [28, 40].

Similarly, data security issues were highlighted by other studies [12, 41, 42]. One of the main challenges for organizations is the security risk of access to the system, privacy, authorization, verification, data and network [33]. Data consistency and integrity become one of the potential challenges when data repeatedly changes, and is shared with numerous collaborators. It is a psychological perception that there is more exposure for hackers to attack in highly interconnected systems [20]. Such fears can be countered only with suitable IT security [36]. A high level of IT expertise and intensive training regarding cybersecurity and privacy issues are also of great advantage. Thus, it is hard to manage sensor-generated information without specific software know-how [12].

Several sources [8, 36] have suggested that a mismatch between i4.0 requirements and the institution’s capacity is also a significant obstacle to implementation. There is a need to adapt the organizational structure for successfully transforming it according to i4.0 requirements [43]. The collaboration between groups and departments, and the provision of sufficient resources are imperative for booming Industry 4.0. There is a common perception about the lack of enough resources among the employees to implement company-wide industry 4.0. It includes both sufficient free human capacities and financial resources. Finding resources and time to focus on the i4.0 implementation is difficult because of the existing tasks of workers. It is, therefore, considered that existing organizational structures are unsuitable regarding IT competencies. The goal of matching i4.0 requirements and the institution’s capacity can only be achieved with patience and sufficient resources [36]. Therefore, the management level should be prepared for disruptive changes. Further, instead of competing, many firms are now collaborating regarding the development of essential infrastructure needed for matching between i4.0 requirements and the institution’s capacity [20], as there is a substantial need to complement it with supplementary parts or services [23].

Likewise, a study by Raj, Dwivedi [20] suggests that the introduction of i4.0 technologies may be significantly affected by the fear of economic loss. Capital of immense amount is required for initiating i4.0 implementation [20], and there are decisions of not investing in new technologies because of perceived barriers [24]. Because high investments regarding technology, processes, and people are required both at the supply chain and corporate levels, most firms are still reluctant to invest in the R&D of i4.0 [20].

Moreover, the profitability of short to medium-term investments can often be negative in i4.0 technologies. There are several reasons behind it: for example, high investments are required along with complex implementations; the benefits occur only with a time lag, and, furthermore, it is hard to measure benefits in financial terms. Eventually, this leads to a trade-off between benefits and costs, efficacy being dependent on a case-by-case company-specific basis.

There is also a high risk regarding the provision of necessary resources. Significant monetary investment in terms of complex machines and systems is required to implement networking technologies and digitization. Because of a time lag between amortization and investment, there are high financial risks on the one hand, and also, these investments are highly uncertain on the other hand [43]. This is exacerbated by the perception that the time-span of i4.0 to result in a beneficial outcome is too long, but companies act to achieve short-term benefits. Consequently, employees think the new product or service does not produce a relative advantage, and could be malfunctioned or dysfunctional [23]. Eventually, management would drag its feet in supporting and leading the change initiative when faced with a lack of experience and resources [44].

Lack of enhanced skills, expertise, and workforce qualification is another barrier to the accelerated development of i4.0 [22, 28]. A psychological barrier in employees is that they do not have the necessary skills to perform their new role, and, therefore, a longer learning time is required [8]. That is why employees do not support the concept of i4.0, as it makes employees afraid of losing their power and influence [43]. Therefore, having a culture that fosters innovation is necessary for harnessing the value of i4.0 [20]. Some other psychological barriers to the adoption of i4.0 are loss of face [45], norm barriers [23], dreaded inequality, i.e., i4.0 will segregate the market into high skills/high pay and low skills/low pay categories [46], realization barriers [3], usage barriers, that is, revolution is incongruent with existing habits [47], and achieving smooth coordination between various organizational departments is also challenging [20]. Kamble, Gunasekaran [22] and Karadayi-Usta [40] identified only 12 and 9 i4.0 barriers, respectively, but other significant obstacles, especially the psychological factors, have not been considered. However, the current study has collated a list of 20 most reported barriers in S1 Appendix to further explore the theme of the psychological obstacles, and the adverse effects they may have on the behavior of agents involved in i4.0 adoption. Based on the currently available literature, S1 Appendix outlines the identification of i4.0 implementation constraints. It is clear that from a psychological point of view that there are three types of barriers: avoidance, authority and misunderstanding [48].

Since the ISM methodology may escalate the complexity with the increasing number of variables [49], the current study, firstly, computed the weights of all the identified twenty barriers through a hybrid AHP-TOPSIS approach. Then ISM methodology was applied to top extracted psychological barriers. The unique contribution of this study is to find not only the significant barriers but also to explore the relationship among them in an effort to allocate scarce organizational resources to them.

## 3. Methodology

**Fig 1:**
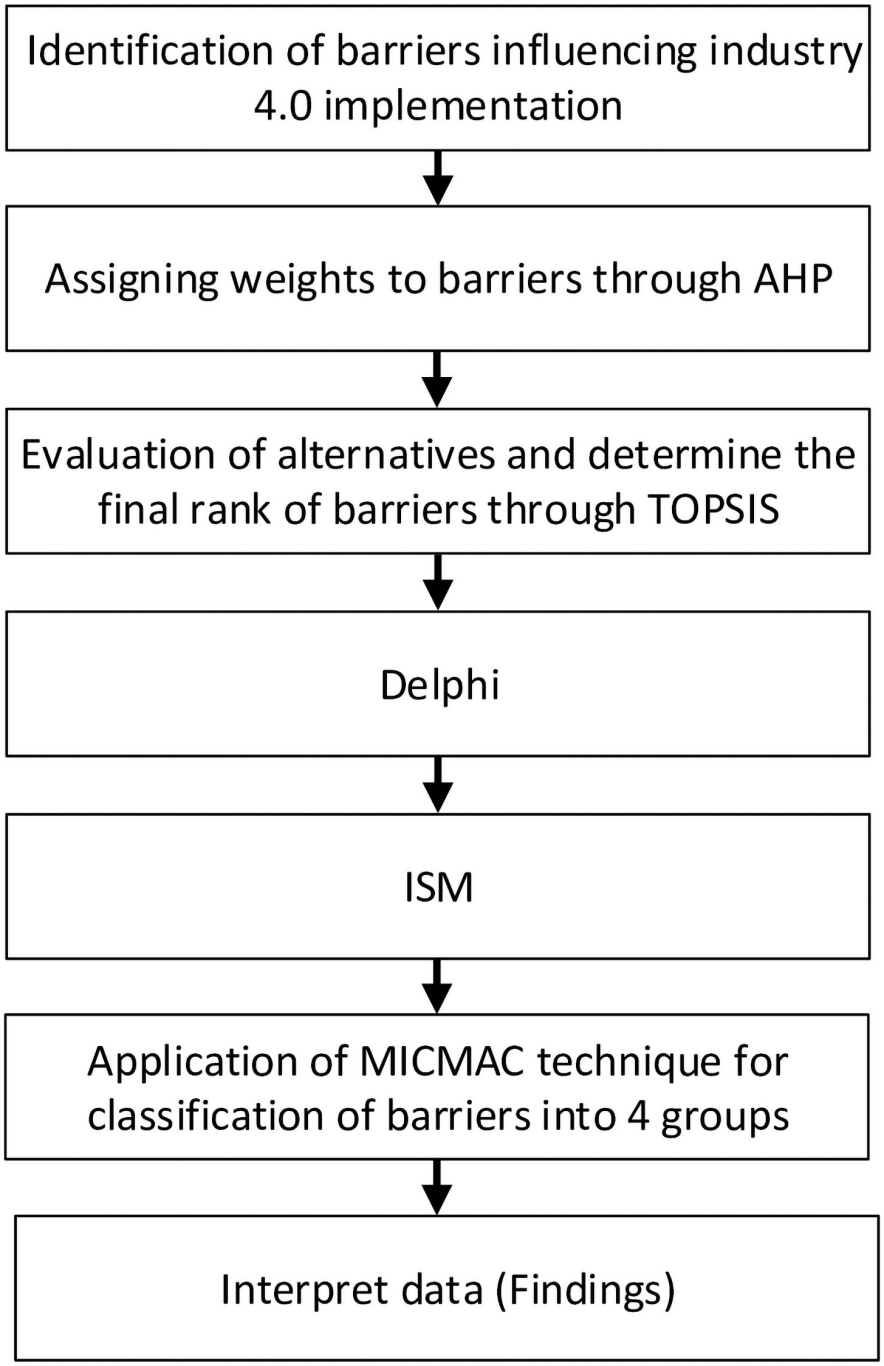
The research process.

In order to analyze the psychological barriers to the implementation of industry 4.0, the proposed methodology comprises four stages. In the first stage, barriers to the successful implementation of i4.0 were identified based on the literature review and experts’ inputs. In the second stage, AHP was applied to determine the relative weights of the barriers, followed by a final ranking of the obstacles through the TOPSIS analysis. In the third stage, relationships between these barriers were analyzed from the perspectives of eight experts employing the ISM technique. In the end, MICMAC analysis was carried out to classify these barriers. Fig 1 depicts our methodological approach.

### 3.1. Sample and procedure

The study has been conducted in the context of future industrial developments. The source of data collection for the current survey was the top-ranked educational institutions and manufacturing sector of Pakistan. To represent the manufacturing organizations where the i4.0 concept has been implemented, the authors purposefully selected state-of-the-art manufacturing organizations from all four major provinces of Pakistan. Another reason for choosing these organizations was that these organizations employ thousands of workers. In order to implement the ISM technique, primary data were obtained through expert elicitation depending on the participant’s knowledge and experience (minimum 10 years) about the topic. Data comprising 105 questions were collected through the Delphi technique for developing contextual relationships between different barriers. In this technique, experts can express their opinions anonymously without the group pressure of social conformity and, hence, minimize the response bias. Furthermore, confidentiality for specific responses is preserved in this strategy [50]. The purposive sampling technique was applied in this study because researchers can choose experts based on their knowledge [51].

There are different opinions about the sample size for the ISM technique. For example, due to the absence of a significant correlation between the number of panel members (experts) and effectiveness [52], recommend a minimum of eight experts. Therefore, most studies engaged the number of panelists between 8 and 16 while using Delphi. Moreover, the panel selection process of experts is crucial because the Delphi method requires extensive experience and knowledge for an efficient evaluation of various alternatives; therefore, Hyun, Cho [53] finally selected seven experts for their study. According to Bolaños, Fontela [54]; Govindan, Azevedo [55], there should not be too many respondents participating in the ISM. Therefore [56], involved five experts answering the questions, while [14] engaged eight experts in their studies. Similarly, Deshpande and Nagendra (2020) invited 13 experts to arrive at the relationships, but only five experts participated. So, in the current study, experienced academicians and managers with more than ten years of experience were invited to elicit expert opinions while using AHP and ISM. Initially, 18 experts were contacted, but 10 responded to participate. However, finally, eight experts participated (three from academia and five from industry), but their views were considered effective for analysis because of their expertise. Three rounds of (Delphi) discussion were carried out to reach an agreement about the interrelations among barriers.

On the other hand, the data for TOPSIS were collected from managers and supervisors of different functional areas of the manufacturing industry in Pakistan. A convenient sampling technique was used to collect data at the operational level of various manufacturing organizations. The questionnaires were distributed to 695 users (managers and supervisors) to rate on a five-point Likert scale varying from “strongly disagree” (1) to “strongly agree” (5). After discarding the incomplete questionnaires, 443 were found completely filled. Thus, the effective response rate was 63.74% of the respondents. The characteristics of the respondents for both studies have been summarized in Table 1.

**Table 1:** Demographics of the respondents.

Technique	Demographic	Frequency	Percent
AHP and ISM	Experts (Having 10+ Years of Experience)		
	Academia	3	37.5
	Industry	5	62.5
TOPSIS	Operational level Users (Managers and Supervisors)		
	Age (Years)		
	25–30	75	17
	31–35	151	34
	36–40	137	31
	Above 40	80	18
	Education (Years)		
	14 Years	372	84
	16 Years	49	11
	18 & Above	22	5

### 3.2. Ethics statement

Ethical review and approval were obtained for this study on human participants from the ethical review committee “Ethical Research Committee” of the Institution (NML-ERC/2020-016). Moreover, the participants provided their written informed consent to participate in this study.

### 3.3. Techniques used in the study

#### 3.3.1. Hybrid AHP-TOPSIS technique for ranking

Several multi-criteria decision-making (MCDM) techniques are used to rank the factors. Some of the popular MCDM methods utilized by many researchers are Analytical Hierarchy Process (AHP), Analytical Network Process (ANP), Technique for Order of Preference by Similarity to Ideal Solution (TOPSIS), Complex Proportional Assessment (COPRAS), Evaluation based on Distance from Average Solution (EDAS), MEthod based on the Removal Effects of Criteria (MEREC), Weighted Aggregated Sum Product Assessment (WASPAS) and Simultaneous Evaluation of Criteria and Alternatives (SECA) [57–62]. One major advantage of AHP over other techniques is that it can convert elements that are intangible and difficult to quantify into quantified and tangible values [53]. Numerous researchers have used the AHP method for prioritization in different fields [63–66].

The relative importance of the factors is assessed through the weights of criteria. Different methods have been introduced to obtain criteria weights, including objective weighting, subjective weighting and hybrid weighting methods. In subjective methods, the preferences of decision-makers determine the criteria weights, but this method is not enough when the number of criteria increases. On the other hand, in objective weighting methods, specific computational processes based on decision-matrix yield criteria weights in which preferences of decision-makers have no role. However, integrating different objective and subjective weighting methods is preferred to combine both characteristics [58]. Hybrid methods give more realistic weights as they can utilize the preference of decision-makers as well as data of the decision matrix [67–69]. Accordingly, the TOPSIS method based on AHP weights (integration of both techniques) has great opportunities as it complements and improves the subjective opinions of decision-makers [69, 70]. Consequently, AHP has been coupled with TOPSIS by many researchers and practitioners, making this combination a success to compute more reliable and error-free results [62, 71–73]. Thus, the current research has integrated these approaches because AHP derives the criteria weights (through selected experts), while TOPSIS facilitates finding the best alternative (through a large number of frontline supervisors) [71]. It is important to note here that AHP was selected instead of ANP because alternatives were considered independent of each other.

**Fig 2:**
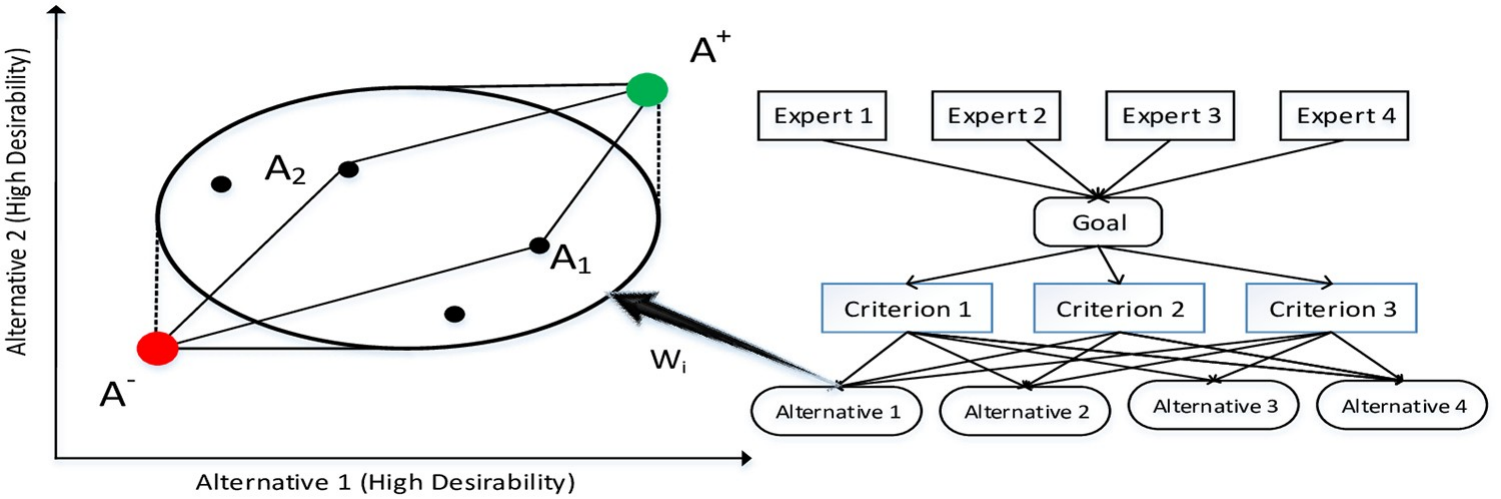
Hybrid AHP-TOPSIS.

Analytical Hierarchy Process (AHP), developed by Satty (1980), is a structured tool to deal with complex, unstructured and multidimensional decision problems. The method is based on the judgment of experts for pair-wise comparisons to alternatives against given multiple criteria in a hierarchical structure (Satty, 2008). It has been applied in many sectors due to its flexibility and robustness. In contrast, TOPSIS (Technique for Order Preference by Similarity to Ideal Solution), developed by Hwang and Yoon (1981), is a multi-criteria decision analysis compensatory aggregation method that allows trade-offs. Monotonically decrease or increase in criteria is the assumption of this method. Whereas, the underlying principle of the technique is that the best alternatives (emerging barriers in this case) should have the largest geometric distance from the negative ideal solution (NIS), whereas the shortest geometric distance from the positive ideal solution (PIS) [74]. The distance is treated as an index value so that the important attributes are closer to unity and the least important is closer to zero, shown in Fig 2.

The relative advantage of TOPSIS is that it quickly identifies the best alternative, requiring minimal subjective input from the decision-makers. The PIS maximizes the benefit criteria and minimizes the cost criteria, whereas NIS does the contrary. It can be expressed that all best values are included in PIS, while all worst values are included in NIS [75].

#### 3.3.2. Interpretive Structural Modeling (ISM) model development

Compared with Graph Theory, Analytical Network Process (ANP), Structural Equation Modeling (SEM), DEMATEL and Fuzzy Cognition Maps, Interpretive Structural Modeling (ISM) methodology is considered well-established to identify the relationships between distinct variables [76]. In Graph Theory, the reliability of the direction of the edges in the graphs is questionable even it reveals the interdependencies among the factors. Based on the cause and effect groups, DEMATEL helps uncover the causal interactions among the factors. Similarly, ANP useful in real-life non-linear problems is less accepted due to its complexity, and SEM ‘a priori’ method requires a large sample size used for the theoretical development of the model [77]. Similarly, in Fuzzy Cognition Maps, the causality reverses to reach a steady-state, and all or some of the Wij become positive if the number of iterations of the algorithm is increased, which is a serious drawback that changes the causality between concepts [78]. Consequently, ISM has a higher ability to capture real-life dynamic complexities [79].

Warfield (1974) proposed the ISM technique to capture the complex interrelationships of emerging socio-economic factors. ISM is an interactive technique used by research groups in complex environments as a communication mode to transfigure ambiguous and equivocal mental models of systems into a well-defined and explicit hierarchical model (Alawamleh & Popplewell, 2011 [80]. ISM has been broadly utilized in the early literature on policymaking to its wide use in recent research [81]. It provides a clear picture of the interrelationships, and presents cooperative identifications of these associations [82].

#### 3.3.3. MICMAC analysis for classification of barriers

Cross-Impact Matrix Multiplication Applied to Classification (MICMAC) analysis (Duperrin & Godet, 1973) is further used to classify complex system elements by reaction paths and loops. The key objective of MICMAC is to analyze the driving and dependence power of each barrier [83]. In many ISM-based studies, the MICMAC technique was used to classify elements into different categories because ISM lacks in exposing the driving and dependence power of elements [84]. Moreover, this clubbed analysis is helpful to differentiate the input, output and intermediate parameters [85]. The elements are categorized into four clusters depending upon their driving power and dependence: Autonomous, Dependent, Linkage and Independent [80].

## 4. A real-case application of the proposed model

The application of the proposed model in manufacturing organizations is to assess the most prominent psychological barriers. The numeric application of all the steps of previously discussed techniques along with the results has been described in this section.

### • Computation of weights

Weights of all the psychological barriers were computed by AHP adopting the steps described in the literature [62, 86, 87].

**Step 1**: From the top through the intermediate level to the bottom level, the decision hierarchy was structured after setting the goal. The first level was about prioritizing the psychological barriers to the i4.0 implementation. In the second level, there were groups of all obstacles based on their attributes. The last level was about alternatives or barriers from which the most influencing barriers had to be selected.
**Step 2**: In this step, experts were solicited using a 1–9 preference scale as described in [88] to create matrices of pair-wise comparisons of all the barriers and groups. This pair-wise comparison is the strength of AHP [3].
**Step 3**: It starts with a mathematical process to normalize each matrix. The eigenvector of a matrix was obtained by taking the average of all values in a normalized matrix row. Then, the principal eigenvalue known as **λ**_**max**_ was obtained through the summation of the product of the eigenvector and the sum of the columns of the reciprocal matrix.
**Step 4**: A consistency test was conducted for the verification of assessment of the decision group using the given formula:
$$CR\;(Consistency\;Ratio)\;=\;\frac{CI}{RI}$$
  where, RI = Random Index, and
$$CI\;(Consistency\;Index)\;=\;\frac{\lambda_{max}-n}{n-1}$$
  Where, n is the matrix size and value of RI was obtained from random inconsistencies index (e.g., for 6×6 matrix, the value of RI = 1.24) [89]. If *CR* > 0.1, it shows that elements are not adequately compared, and a review is required.

In the current study, a three-level hierarchical structure was developed from different psychological barriers concerning AHP analysis. Unlike the conventional structure in most AHP models, there was a goal of identifying the most prominent psychological barriers at the first level. The experts made four groups of all the barriers placed at the criteria level: **Idiosyncratic, Extramural, Transference and Consternation**, as shown in Table 2. The full description of the notations used for barriers has been provided in S1 Appendix. The psychological barriers were at the third or sub-criteria level, while there was no alternative at the bottom. Pair-wise comparisons of barriers and groups were made by keeping in mind the objective using the fundamental preference scale of absolute numbers. As all the CR values were less than 0.1, it showed that all the comparison matrices were of good consistency. The assigned and normalized weights of the barriers representing the relative causal importance of each barrier itself were obtained through experts’ judgment, as shown in the second last column of Table 2.

**Table 2:** The importance weights of all barriers (comparison of priority vectors of all the questionnaires).

Barriers	Expert 1	Expert 2	Expert 3	Expert 4	Expert 5	Expert 6	Expert 7	Expert 8	Relative Preference Weights (Geometric Mean)	Global Preference weights
**Group 1 (Idiosyncratic Barriers)**									**0.5022**	
B1	0.2361	0.2175	0.2468	0.2229	0.2264	0.2373	0.2364	0.2373	0.2324	0.1167
B2	0.1158	0.1266	0.0985	0.0600	0.0617	0.0633	0.0610	0.0635	0.0776	0.0390
B3	0.1623	0.1566	0.1447	0.1100	0.1108	0.1153	0.1124	0.1164	0.1270	0.0638
B8	0.0824	0.0866	0.0959	0.1498	0.1588	0.1645	0.1898	0.1692	0.1309	0.0657
B13	0.0506	0.0439	0.0516	0.0915	0.0952	0.1006	0.1026	0.1183	0.0769	0.0386
B17	0.353	0.3688	0.3624	0.3658	0.3470	0.3190	0.2978	0.2953	0.3374	0.1694
C.I	0.0834	0.0774	0.0687	0.0991	0.1051	0.0936	0.1163	0.1232	0.0942	
C.R	0.0667	0.0619	0.0550	0.0793	0.0841	0.0749	0.0931	0.0986	0.0754	
**Group 2 (Extramural Barriers)**									**0.2631**	
B5	0.2667	0.2169	0.2585	0.2273	0.2197	0.2314	0.2344	0.2499	0.2375	0.0625
B4	0.0616	0.1263	0.1018	0.0604	0.0623	0.0645	0.0634	0.0611	0.0723	0.0190
B12	0.1030	0.1562	0.1513	0.1124	0.1133	0.1195	0.1055	0.1011	0.1187	0.0312
B18	0.1083	0.0468	0.0538	0.0994	0.0994	0.1085	0.1233	0.1052	0.0886	0.0233
B19	0.3191	0.3676	0.3363	0.3476	0.3420	0.3051	0.2987	0.3392	0.3313	0.0872
B20	0.1412	0.0862	0.0984	0.1530	0.1633	0.1710	0.1747	0.1436	0.1376	0.0362
C.I	0.0969	0.0989	0.0466	0.0980	0.0893	0.0719	0.0658	0.0994	0.0809	
C.R	0.0775	0.0791	0.0373	0.0784	0.0715	0.0575	0.0527	0.0795	0.0647	
**Group 3 (Transference Barriers)**									**0.1426**	
B7	0.0901	0.1098	0.1243	0.1111	0.1113	0.0910	0.0848	0.1120	0.1035	0.0148
B14	0.5462	0.3104	0.4242	0.5205	0.5402	0.5234	0.5983	0.4132	0.4756	0.0678
B15	0.0945	0.0571	0.0778	0.0770	0.0840	0.0897	0.0811	0.0717	0.0783	0.0112
B16	0.2691	0.5227	0.3736	0.2915	0.2645	0.2959	0.2358	0.4030	0.3214	0.0458
C.I	0.0254	0.0894	0.0699	0.0247	0.0592	0.0072	0.0327	0.0644	0.0370	
C.R	0.0282	0.0994	0.0776	0.0274	0.0657	0.0080	0.0364	0.0715	0.0411	
**Group 4 (Consternation Barriers)**									**0.0921**	
B11	0.1601	0.1557	0.1507	0.1503	0.1572	0.1118	0.1089	0.1151	0.1371	0.0126
B6	0.4532	0.4787	0.4566	0.4363	0.4597	0.4118	0.3942	0.3591	0.4295	0.0396
B9	0.0817	0.0739	0.0784	0.0787	0.0886	0.0737	0.0707	0.0756	0.0775	0.0071
B10	0.3050	0.2918	0.3142	0.3347	0.2945	0.4027	0.4262	0.4502	0.3476	0.0320
C.I	0.0400	0.0398	0.0705	0.0716	0.0153	0.0243	0.0708	0.0242	0.0388	
C.R	0.0445	0.0442	0.0784	0.0795	0.0170	0.0270	0.0787	0.0269	0.0431	

### 4.2. Determination of final ranking

In order to determine the final ranking, a questionnaire was developed based on 20 psychological barriers while using TOPSIS in the current study. It involved collecting firm-level data from managers and supervisors who are actually in the process of digitalization. The authors followed the TOPSIS steps: first, a decision matrix (***x***_***ij***_) was prepared based on information provided by users, which illustrates the importance of different causal barriers. Then, the decision matrix was normalized (***r***_***ij***_) as:

$$r_{ij}\;=\;\frac{x_{ij}}{\sqrt{\sum x_{ij}^{2}}}$$ (1)

where, *i* = 1,….., *m* and *j* = 1,……, *n*

***x***_***ij***_ = original decision matrix and

***r***_***ij***_ = normalized decision matrix

After that, a weighted normalized decision matrix was constructed by multiplying each column of the matrix ***r***_***ij***_ with weight *w*_*j*_.

$$\nu_{ij}\;=\;r_{ij}\times w_{j}$$ (2)

for *i* = 1,….., *m* and *j* = 1,……, *n*

Where *w*_*j*_ is the weight acquired from AHP in the current study. The computation of the weighted normalized matrix using Eq (2) is given in Table 3.

**Table 3:** Weighted normalized decision matrix.

Barriers	*V*5*j*	*V*4*j*	*V*3*j*	*V*2*j*	*V*1*j*
B1	0.0915	0.0548	0.0443	0.0167	0.0038
B2	0.0015	0.0149	0.0286	0.0139	0.0169
B3	0.0101	0.0308	0.0530	0.0109	0.0098
B4	0.0072	0.0093	0.0043	0.0012	0.0008
B5	0.0451	0.0403	0.0145	0.0053	0.0017
B6	0.0184	0.0277	0.0212	0.0031	0.0013
B7	0.0101	0.0093	0.0052	0.0014	0.0005
B8	0.0513	0.0309	0.0245	0.0105	0.0050
B9	0.0038	0.0044	0.0040	0.0008	0.0007
B10	0.0046	0.0161	0.0261	0.0065	0.0046
B11	0.0593	0.0466	0.0322	0.0077	0.0027
B12	0.0319	0.0255	0.0180	0.0043	0.0034
B13	0.0290	0.0218	0.0124	0.0041	0.0006
B14	0.0387	0.0419	0.0352	0.0088	0.0053
B15	0.0075	0.0071	0.0039	0.0014	0.0005
B16	0.0271	0.0332	0.0148	0.0062	0.0019
B17	0.1375	0.0783	0.0565	0.0197	0.0095
B18	0.0010	0.0102	0.0194	0.0078	0.0102
B19	0.0687	0.0416	0.0317	0.0113	0.0028
B20	0.0042	0.0327	0.0636	0.0331	0.0370

Furthermore, there is identification of positive ideal solution V+ and negative ideal solution V-. Positive ideals and negative ideals denoted by *A*_+_ and *A*_−_, respectively are the maximum and minimum values in each column of the weighted normalized decision matrix.


$$A_{+}\;=\;\left\{\left(max\left(\nu_{ij}|\;i\;=\;1,2,\ldots ,m\right)\left|j\in J_{-}\right.\right),\;\left(min\left(\nu_{ij}|i\;=\;1,2,\ldots ,m\right)\left|j\in J_{+}\right.\right)\right\}$$ (3)

$$A_{-}\;=\;\left\{\left(min\left(\nu_{ij}|\;i\;=\;1,2,\ldots ,m\right)\left|j\in J_{+}\right.\right),\;\left(max\left(\nu_{ij}|i\;=\;1,2,\ldots ,m\right)\left|j\in J_{-}\right.\right)\right\}$$ (4)

Eqs (3) to (4) were used to determine the positive and negative ideal solutions as shown in Table 4.

**Table 4:** Ideal best and negative ideal solution matrix.

	Ideal (Best) A+		Negative-ideal A-
Vi1^**+**^	0.1375	Vi1^**-**^	0.0010
Vi2^**+**^	0.0783	Vi2^**-**^	0.0044
Vi3^**+**^	0.0565	Vi3^**-**^	0.0039
Vi4^**+**^	0.0197	Vi4^**-**^	0.0008
Vi5^**+**^	0.0169	Vi5^**-**^	0.0005

Now for each alternative, separation measure was calculated here. Separation is calculated from positive ideal alternative as:

$$S_{+}^{i}\;=\;\sqrt{\sum_{j\;=\;1}^{m}{\left(\nu_{ij}-\nu_{j}^{+}\right)}^{2}},\;i\;=\;1,2,3\ldots ,m$$ (5)

$S_{+}^{i}$ stands for distance *i* from the positive ideal.

From negative ideal alternative, calculation of separation is as

$$S_{-}^{i}\;=\;\sqrt{\sum_{j\;=\;1}^{m}{\left(\nu_{ij}-\nu_{j}^{-}\right)}^{2}},\;i\;=\;1,2,3\ldots ,m$$ (6)

$S_{-}^{i}$ stands for distance *i* from the negative ideal

$$C_{i}\;=\;\frac{S_{-}^{i}}{S_{-}^{i}+S_{+}^{i}}$$ (7)

The Euclidean separation distances were calculated using Eqs (5) and (6). After determining the distance from a positive and negative ideal solution, there has been computation of relative closeness (Ci) to the ideal solution of each barrier by using Eq (7). Table 5 summarizes the results, where the distance to the ideals, relative closeness and the proposed rank can be seen.

**Table 5:** Euclidean separation distance.

	Si+	Distance i from positive Ideal	Si-	Distance i from Negative-ideal	Relative closeness (Ci) values	The proposed rank
B1	S1+	0.0548	S1-	0.1123	0.6721	2
B2	S2+	0.1527	S2-	0.0341	0.1823	14
B3	S3+	0.1365	S3-	0.0581	0.2984	9
B4	S4+	0.1583	S4-	0.0079	0.0478	18
B5	S5+	0.1104	S5-	0.0581	0.3448	8
B6	S6+	0.1361	S6-	0.0339	0.1995	13
B7	S7+	0.1557	S7-	0.0104	0.0628	17
B8	S8+	0.1046	S8-	0.0614	0.3699	5
B9	S9+	0.1635	S9-	0.0028	0.0168	20
B10	S10+	0.1510	S10-	0.0263	0.1484	15
B11	S11+	0.0898	S11-	0.0777	0.4638	4
B12	S12+	0.1258	S12-	0.0403	0.2426	10
B13	S13+	0.1320	S13-	0.0342	0.2060	12
B14	S14+	0.1086	S14-	0.0624	0.3648	7
B15	S15+	0.1592	S15-	0.0071	0.0428	19
B16	S16+	0.1280	S16-	0.0408	0.2416	11
B17	S17+	0.0074	S17-	0.1652	0.9571	1
B18	S18+	0.1576	S18-	0.0205	0.1150	16
B19	S19+	0.0834	S19-	0.0828	0.4982	3
B20	S20+	0.1431	S20-	0.0822	0.3648	6

Barriers with the maximum C_i_ values were the major prominent and causal psychological barriers to the i4.0 implementation, as shown by the TOPSIS calculation. Psychological barriers can be ranked as B17-B1-B19-B11-B8-B20-B14-B5-B3-B12-B16-B13-B6-B2-B10-B18-B7-B4-B15-B9 in the decreasing order of preference. From Table 5, it is clear that there is a significant influence of the weighting factor on the ranking order.

### 4.3. Causal model of barriers

ISM model was developed by extracting the top 15 barriers from Table 5 because the increased variables may escalate the complexity of ISM methodology [49]. These selected barriers were renumbered and recoded from B (barrier) to BM (barrier for model). The use of the ISM technique is appropriate because the representation and interpretation of the interactions among 15 barriers (considerably large) require a well-established modeling approach [90].

ISM is a holistic social system engineering technique used to model multilevel hierarchical structural cross-domain relationships based on mathematical operations. As the structural level increases, the complexity level intensifies accordingly. The following procedure has been adopted to construct the model (Singh, & Kant, 2008).

Suppose there are n elements in a system set S so that *S* = {*s*_1_, *s*_2_, *s*_3_, …, *s*_*n*,_}, and its cross product is *S* × *S* = {(*s*_*i*_, *s*_*j*_)│*s*_*i*_, *s*_*j*_ ∈ *S*}, that would satisfy conditions of transitivity, symmetry and reflexivity. The specific brief steps are as follows:

Step 1: Structural self-interaction matrix (SSIM) development
Step 2: Compute the reachable matrix
Step 3: Transform reachable matrix to hierarchical matrix (Level partitions)

Finally, the ISM relationships diagram is drawn.

#### Step 1: Structural self-interaction matrix (SSIM) development

A value V, A, X or O was used in the structured questionnaire to categorize the relationships between any two barriers according to the below-given rules:

V = barrier i affects barrier j
A = barrier j affects barrier i
X = interrelationship between (i, j) barriers mean both are helpful to achieve each other
O = no relationship between (i, j) barriers

A contextual relationship was developed between each pair of elements using experts’ opinions based on the Delphi technique. Respondents filled the questionnaires by comparing the column statement of 105 questions to the row statement. The questions were about the direct link between barriers *i* and *j*. SSIM has been developed based on these relationships that emerged from the final results of Delphi round III, as shown in Table 6.

**Table 6:** Structural self-interaction matrix.

Barriers	BM1	BM2	BM3	BM4	BM5	BM6	BM7	BM8	BM9	BM10	BM 11	BM 12	BM 13	BM 14	BM 15
Lack of continued education of employees (BM1)	**1**	X	X	X	V	V	V	V	V	V	V	V	V	V	V
Fear of job losses/ Employment disruptions (BM2)		**1**	X	X	V	V	V	V	V	V	A	V	V	V	V
Lack of standards and reference architecture (BM3)			**1**	V	V	V	V	V	V	V	V	V	V	V	V
Fear of data loss/Risk of security breaches (BM4)				**1**	V	V	V	V	V	V	V	V	V	V	V
Lack of necessary talent (BM5)					**1**	V	X	X	X	X	X	V	V	V	V
Challenges in value-chain integration (BM6)						**1**	A	A	A	A	A	A	A	A	X
No venturing motivation (BM7)							**1**	X	X	X	X	V	V	V	V
Compatibility BMarrier (BM8)								**1**	X	X	X	V	V	V	V
Loss of face/image (BM9)									**1**	V	X	V	V	X	V
Usage Barriers (BM10)										**1**	X	V	V	V	V
Lack of a leader with appropriate skills, competencies and experience (BM11)											**1**	V	V	V	V
Personality/Low tolerance for change (BM12)												**1**	X	X	V
Fear of economic loss (BM13)													**1**	X	V
Fear of outdatedness of competency (BM14)														**1**	V
Realization Barrier/Uncertainty (BM15)															**1**

#### Step 2: Compute the reachable matrix

- **Initial Reachability Matrix (IRM) Formation**
  After that, the initial reachability matrix (IRM) was developed by replacing V, A, X and O with 1s and 0s using Eq (8), as displayed in Table 7.
i,j=V,i,j=1,j,i=0;A,i,j=0,j,i=1;X,i,j=1,j,i=1;O,i,j=0,j,i=0; (8)
- **Final Reachability Matrix Formation**
  The final reachability matrix (FRM) was developed by incorporating the transitivity represented by 1*. An elementary assumption of ISM is that if element X is related to Y and element Y is connected with Z, it means X is necessarily related to Z. FRM was computed according to the Boolean rules below given.
$$R\;=\;{\left(A+I\right)}^{r}\;=\;{\left(A+I\right)}^{r-1\;}\neq {\left(A+1\right)}^{r-2}\neq \ldots \;\neq \left(A+1\right),\;r\leq 14$$ (9)
  In Eq (9), the final reachability matrix has been shown by *R*; *r* represents less than or equal to the number of barriers, *I* is the unit matrix, and *A* represents the initial reachability matrix. In this step, there is also the identification of the driving and dependence power of each barrier. The sum of 1s in a given column computes the dependence power of a barrier. Whereas, the sum 1s in a row is the driving power of that particular barrier. Table 8 shows the final reachability matrix.

**Table 7:** Initial reachability matrix.

Barriers	B1	B2	B3	B4	B5	B6	B7	B8	B9	B10	B11	B12	B13	B14	B15	Driving Power
B1	1	1	1	1	1	1	1	1	1	1	1	1	1	1	1	15
B2	1	1	1	1	1	1	1	1	1	1	0	1	1	1	1	14
B3	1	1	1	1	1	1	1	1	1	1	1	1	1	1	1	15
B4	1	1	0	1	1	1	1	1	1	1	1	1	1	1	1	14
B5	0	0	0	0	1	1	1	1	1	1	1	1	1	1	1	11
B6	0	0	0	0	0	1	0	0	0	0	0	0	0	0	1	2
B7	0	0	0	0	1	1	1	1	1	1	1	1	1	1	1	11
B8	0	0	0	0	1	1	1	1	1	1	1	1	1	1	1	11
B9	0	0	0	0	1	1	1	1	1	1	1	1	1	1	1	11
B10	0	0	0	0	1	1	1	1	0	1	1	1	1	1	1	10
B11	0	1	0	0	1	1	1	1	1	1	1	1	1	1	1	12
B12	0	0	0	0	0	1	0	0	0	0	0	1	1	1	1	5
B13	0	0	0	0	0	1	0	0	0	0	0	1	1	1	1	5
B14	0	0	0	0	0	1	0	0	1	0	0	1	1	1	1	6
B15	0	0	0	0	0	1	0	0	0	0	0	0	0	0	1	2
Dep. Power	4	5	3	4	10	15	10	10	10	10	9	13	13	13	15	144

**Table 8:** Final reachability matrix.

Barriers	B1	B2	B3	B4	B5	B6	B7	B8	B9	B10	B11	B12	B13	B14	B15	Driving Power
B1	1	1	1	1	1	1	1	1	1	1	1	1	1	1	1	15
B2	1	1	1	1	1	1	1	1	1	1	1*	1	1	1	1	15
B3	1	1	1	1	1	1	1	1	1	1	1	1	1	1	1	15
B4	1	1	1*	1	1	1	1	1	1	1	1	1	1	1	1	15
B5	0	0	0	0	1	1	1	1	1	1	1	1	1	1	1	11
B6	0	0	0	0	0	1	0	0	0	0	0	0	0	0	1	2
B7	0	0	0	0	1	1	1	1	1	1	1	1	1	1	1	11
B8	0	0	0	0	1	1	1	1	1	1	1	1	1	1	1	11
B9	0	0	0	0	1	1	1	1	1	1	1	1	1	1	1	11
B10	0	0	0	0	1	1	1	1	1*	1	1	1	1	1	1	11
B11	1*	1	1*	1*	1	1	1	1	1	1	1	1	1	1	1	15
B12	0	0	0	0	0	1	0	0	0	0	0	1	1	1	1	5
B13	0	0	0	0	0	1	0	0	0	0	0	1	1	1	1	5
B14	0	0	0	0	0	1	0	0	1	0	0	1	1	1	1	6
B15	0	0	0	0	0	1	0	0	0	0	0	0	0	0	1	2
Dep. power	5	5	5	5	10	15	10	10	11	10	10	13	13	13	15	150

#### Step 3: Transform reachable matrix to hierarchical matrix (level partitions)

The reachability set R(Pi) and antecedents set A(Pi) for each barrier were obtained from FRM. Reachability set R(Pi) is composed of a particular barrier and the other that it may help achieve, whereas antecedent set A(Pi) comprises a specific barrier itself and the other barriers that may influence it. The intersection of corresponding R(Pi) and A(Pi) represent the intersection set C(Pi) for all barriers. Where, R(Pi) = C(Pi) is the top-level position barrier because it does not help achieve any other barrier above its own level. Then, there is to remove that top-level identified barrier from further analysis. The process is iterated to determine the level of all the barriers. These levels help build a digraph, and final ISM-based model structure. Table 9 showed the hierarchical levels of all barriers.

**Table 9:** Hierarchical division of barriers.

Barriers	*R*(Pi)	*A*(Pi)	*C*(Pi)	Level
BM6	6,15	1,2,3,4,5,6,7,8,9,10,11,12,13,14,15	6,15	*I*
BM15	6,15	1,2,3,4,5,6,7,8,9,10,11,12,13,14,15	6,15	*I*
BM12	12,13,14	1,2,3,4,5,7,8,9,10,11,12,13,14	12,13,14	*II*
BM13	12,13,14	1,2,3,4,5,7,8,9,10,11,12,13,14	12,13,14	*II*
BM14	9,12,13,14	1,2,3,4,5,7,8,9,10,11,12,13,14	9,12,13,14	*II*
BM5	5,7,8,9,10,11	1,2,3,4,5,7,8,9,10,11	5,7,8,9,10,11	*III*
BM7	5,7,8,9,10,11	1,2,3,4,5,7,8,9,10,11	5,7,8,9,10,11	*III*
BM8	5,7,8,9,10,11	1,2,3,4,5,7,8,9,10,11	5,7,8,9,10,11	*III*
BM9	5,7,8,9,10,11	1,2,3,4,5,7,8,9,10,11	5,7,8,9,10,11	*III*
BM10	5,7,8,9,10,11	1,2,3,4,5,7,8,9,10,11	5,7,8,9,10,11	*III*
BM11	1,2,3,4,5,7,8,9,10,11	1,2,3,4,5,7,8,9,10,11	1,2,3,4,5,7,8,9,10,11	*III*
BM1	1,2,3,4	1,2,3,4	1,2,3,4	*IV*
BM2	1,2,3,4	1,2,3,4	1,2,3,4	*IV*
BM3	1,2,3,4	1,2,3,4	1,2,3,4	*IV*
BM4	1,2,3,4	1,2,3,4	1,2,3,4	*IV*

**Fig 3:**
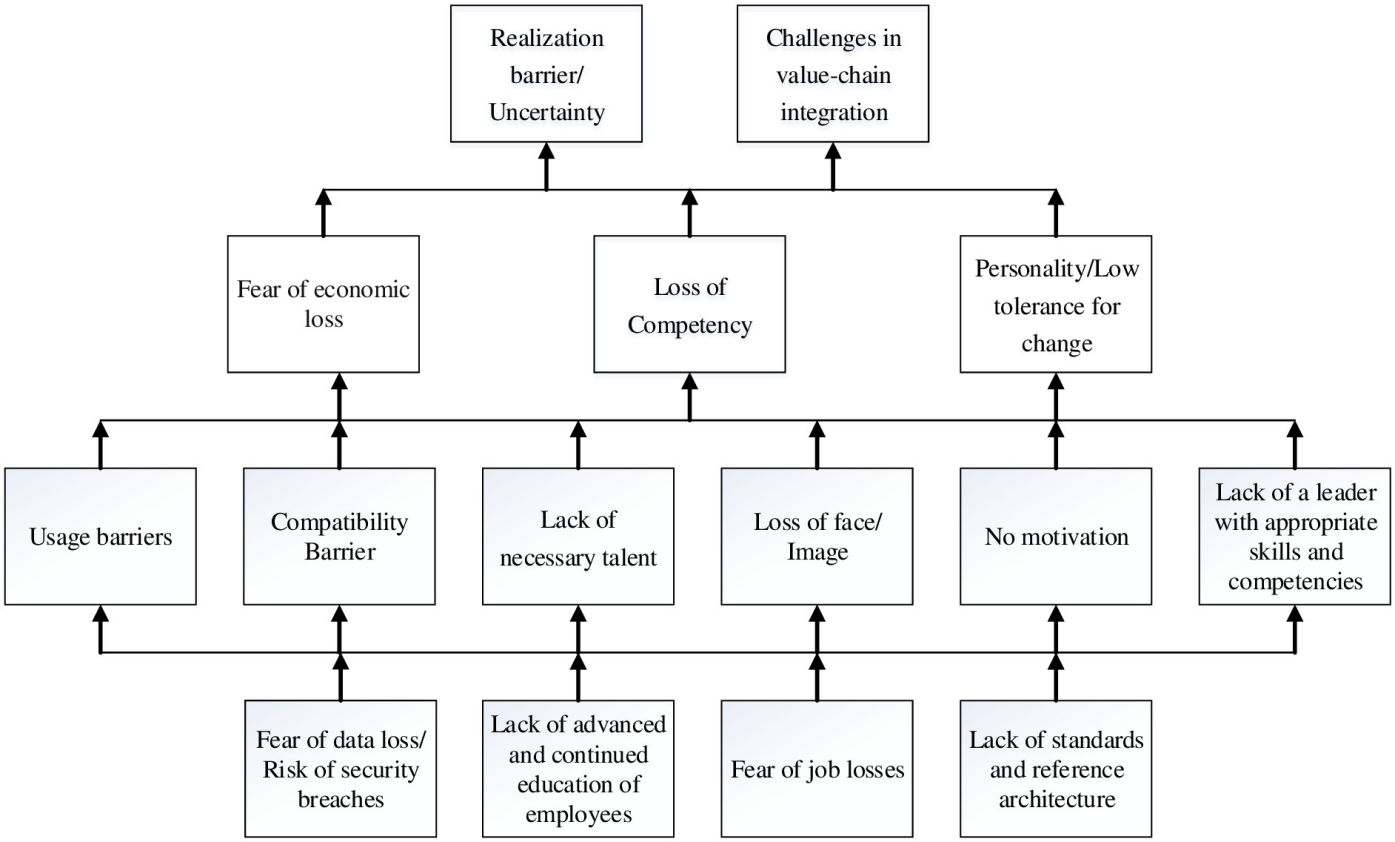
The hierarchical structure of barriers.

A digraph was drawn based on the above-defined relationships (not shown here for brevity). Each arrow in this structure represents “leads to” and the bottom to top approach was used. The digraph was converted to an interpretive structural model of barriers by removing transitivity links and replacing the element nodes of the digraph with statements, as shown in Fig 3. The barriers at the same level have been placed in the same layer.

### 4.4. Clustering of barriers (MICMAC analysis)

**Fig 4:**
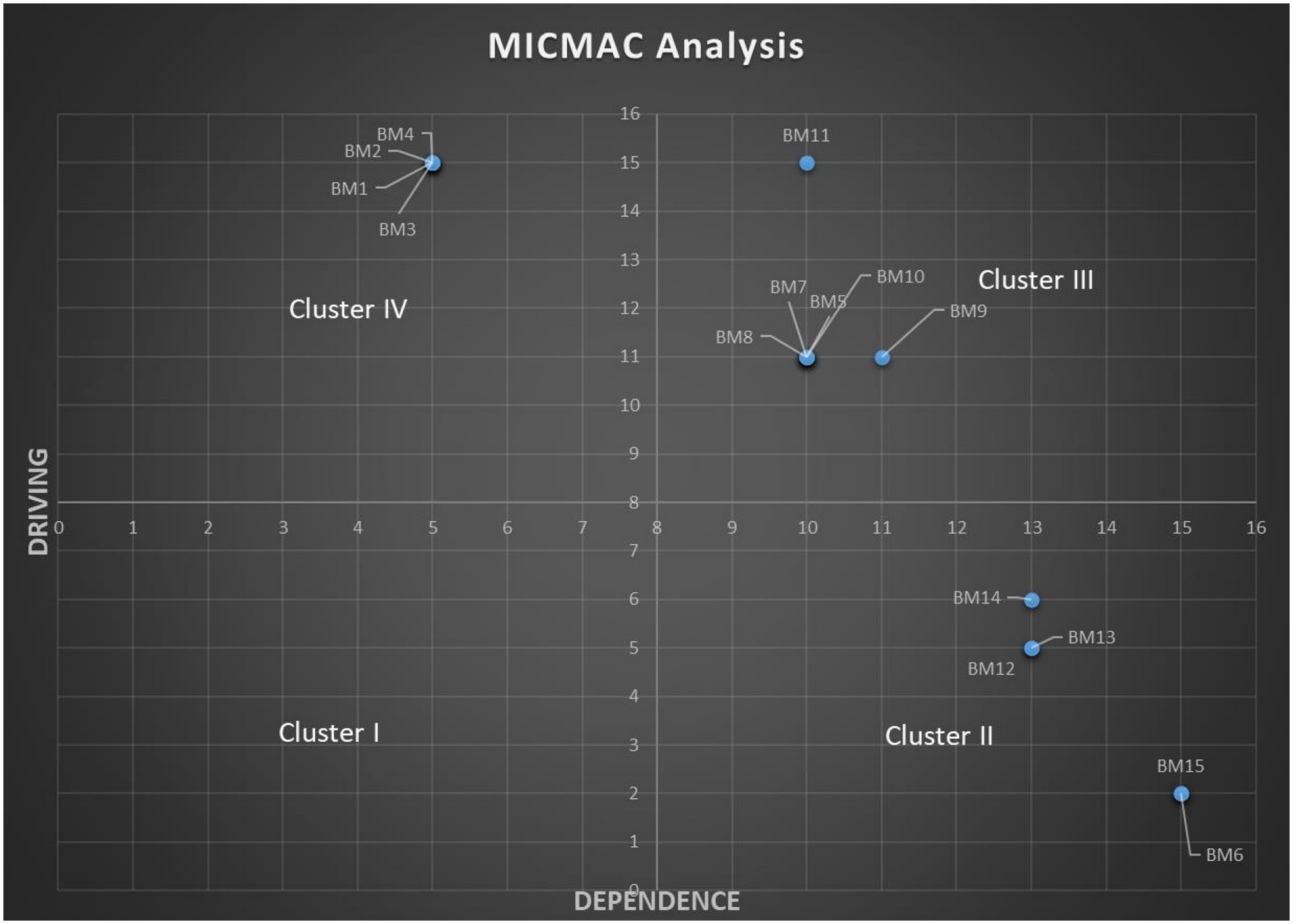
Driver power–dependence diagram.

An indirect classification technique known as MICMAC analysis was used to investigate the scope of each barrier. The diagram was constructed by plotting the driving power on Y-axis and dependence power on X-axis, as shown in Fig 4. This figure demonstrates the distribution of barriers from the perspectives of driving power and dependence power.

The barriers have been classified into four clusters: 1. Autonomous (Cluster I), 2. Dependent (Cluster II), 3. Linkage (Cluster III), and 4. Independent (Cluster IV).

1. Autonomous barriers with weak driving and dependence powers are in Cluster I. Such barriers are considered to be detached and out of the system because of weak links among them. The autonomous cluster was empty because all the identified barriers are highly interrelated in the current case. Therefore, management should pay serious attention to all obstacles to the adoption of industry 4.0.
2. Cluster II is about dependent barriers with weak driving but strong dependence power. Their development mainly occurs based on other barriers. Such barriers exhibit attributes of outcome variables, and are plotted in the right-bottom portion of the graph. Five barriers, viz., low tolerance for change (BM12), fear of economic loss (BM13), loss of competency (BM14), challenges in value-chain integration (BM6) and realization barrier (BM15), have been placed in this cluster.
3. Linkage factors with strong driving and strong dependence powers constitute Cluster III. They are plotted in the right-top portion of the graph because their values are above the middle of both scales. Barriers in the linkage cluster are unstable because linkage variables affect themselves as well as others. Such barriers not only dependent on other barriers but also drive the system. In our study, we find six barriers, viz., ‘Lack of necessary talent (BM5)’, ‘No venturing motivation (BM7)’, ‘Compatibility Barrier (BM8)’, ‘Loss of face/image (BM9)’, ‘Usage barriers (BM10)’, ‘Lack of a leader with appropriate skills’ and ‘competencies and experience (BM11)’ falling in this cluster.
4. Independent sfactors, referred to as driver barriers, constitute the last Cluster IV with weak dependence power but strong driving power. They are in the left-top portion of the graph. Their improvement would have a ripple effect because they influence all the other obstacles in the system significantly. In our study, ‘Lack of continued education of employees (BM1)’, ‘Fear of job losses/ Employment disruptions (BM2)’, ‘Lack of standards and reference architecture (BM3)’ and ‘Fear of data loss/Risk of security breaches (BM4)’ were identified as the driving barriers.

## 5. Discussion

The results of the analyses have been summarized in the previous section in Figs 3 and 4. Interactive relationships between the barriers have been represented in Fig 3, while the distribution of these barriers from the perspective of driving power and dependence power has been displayed in Fig 4. The “driving barriers” can be observed at the bottom level of the hierarchical structure from Fig 3. It includes ‘Lack of continued education of employees (BM1)’, ‘Fear of job losses/ Employment disruptions (BM2)’, ‘Lack of standards and reference architecture (BM3)’ and ‘Fear of data loss/Risk of security breaches (BM4)’. This suggests that priority measures and actions should be taken for these barriers. The findings corroborate the previous studies, for example, “Lack of advanced and continued education of employees” is the main cause of the i4.0 implementation challenges and triggers other barriers [28, 40, 91]. The findings are also aligned with Kamble, Gunasekaran [22], which states that Fear of job losses and Fear of data loss represents the harmonized behavior of being highly influential. Furthermore, “Lack of standards and reference architecture” is another important driving barrier, and these results are in agreement with [17]. A previous study by Raj, Dwivedi [20] is also consistent with this established result. On the same track, the current study appeared to meet with what came along with Kumar, Suhaib [3], who argue that lack of standard and data security concerns are the driving barriers.

Linkage barriers classified in Fig 4 are: ‘Lack of necessary talent (BM5)’, ‘No venturing motivation (BM7)’, ‘Compatibility Barrier (BM8)’, ‘Loss of face/image (BM9)’, ‘Usage barriers (BM10)’, ‘Lack of a leader with appropriate skills’ and ‘competencies and experience (BM11)’. They are positioned at the intermediate level in the hierarchical structure in Fig 3. They receive the influence from the driving barriers and, in turn, exert effects on dependent barriers. BM5, BM7, BM8, BM9, BM10 and BM11 will be triggered and affected by driving barriers (BM1, BM2, BM3 and BM4). The literature also suggests that BM1 (Lack of advanced and continued education of employees) influences BM5 (Lack of necessary talent) and BM12 (Personality/Low tolerance for change) [40]. The findings are also aligned with Kamble, Gunasekaran [22], which states that BM8 (Mismatch between i4.0 requirements and the institution’s capacity/ Compatibility Barrier) and BM5 (Lack of necessary talent and enhanced skills) represent the harmonized behavior of being highly influent and highly dependent.

The linkage barriers, BM5, BM7, BM8, BM9, BM10 and BM11, will exert influence on the dependent barriers such as low tolerance for change (BM12), fear of economic loss (BM13), loss of competency (B14), challenges in value-chain integration (BM6) and realization barrier (BM15). Within the entire system, these barriers exhibit the attributes of outcome variables. The findings of Kamble, Gunasekaran [22] also indicate that BM13 and BM15 are highly driven and dependent on the other input variables included in the system. These findings also align with Raj, Dwivedi [20], who state that there is considerable uncertainty and fear of economic loss due to high investments in i4.0 implementation. Accordingly, we can also interpret that all defined problems can cause an interruption in the i4.0 transformation. Therefore, above stated, all the psychological barriers must be taken into account while moving toward digitalization.

### 5.1. Theoretical and managerial implications

Since it is a relatively novel concept in developing countries, i4.0 requires more elaborative studies to draw clear interpretations and definitions [31]. Academicians are encouraged to categorize other crucial issues to achieve sustainable benefits. I4.0 offers stability to the processes through the digital management of the manufacturing process [92] and removing the identified hurdles will improve economic performance and reduce manufacturing costs. In the current study, the methodological and theoretical foundation was laid to the identified psychological barriers using AHP, TOPSIS, ISM and MICMAC. In doing so, the current research work is one of the preliminary contributions in developing interplay among the psychological barriers of i4.0.

i4.0 is believed to provide efficient resource allocation based on real-time information, and thus, practitioners can benefit from sustainable practices by using i4.0 technologies [93]. The findings of the current study offer several implications for industry practitioners. They can minimize the effect of identified 20 psychological barriers for the successful implementation of Industry 4.0. Further, the relationship among the identified obstacles and their classification based on their driving and dependence power would help them efficiently utilize the scarce resources. The study would also support policymakers and practitioners in understanding the i4.0 adoption process and the barriers hindering its implementation. In this way, it would allow an efficacious performance of i4.0 in the manufacturing industry by overcoming the identified obstacles, making the manufacturing systems more economical and flexible. The policymakers and practitioners may also utilize the developed ISM framework in order to understand the inter-relationships between the barriers in building a valid and operative digital manufacturing platform. It is observed from the study that “Lack of continued education of employees (BM1)’, ‘Fear of job losses/ Employment disruptions (BM2)’, ‘Lack of standards and reference architecture (BM3)’ and ‘Fear of data loss/Risk of security breaches (BM4)” barriers hold utmost importance as they directly or indirectly control every other barrier. So, finally, the practitioners can extrapolate this model to track down the obstacles that need more attention.

## 6. Conclusion

Twenty key i4.0 adoption psychological barriers were identified using an extensive literature survey, and validated by industry experts and frontline managers of the manufacturing sector. Three data analysis techniques (AHP, TOPSIS and ISM) have been uniquely implemented to study i4.0 adoption psychological barriers. It attempts to highlight the most significant and driver barriers, and overcome them, which can act as a major impetus in adopting i4.0. They were classified into four categories based on their driving and dependence powers. Independent barriers are of crucial significance because of their high driving power and less dependence. Finally, a novel causal model was developed with the help of top-listed psychological barriers to facilitate the root cause analysis.

The findings in terms of the dominant impact of these identified barriers corroborate with several previous investigations (for example, [20, 22, 28, 40, 91]). The study is invaluable for policymakers and practitioners in understanding the i4.0 adoption process and the barriers hindering its implementation. However, the present research some limitations, which can be considered future directions. Future researchers should replicate the study to other than the manufacturing sector. Data should be collected from other regions employing longitudinal research design. Moreover, the final model should be validated statistically through Structural Equation Modeling (SEM) technique.

## 7. Limitations and future directions

Despite several contributions of the study, it has some limitations that would further open horizons for future researchers. First, since the driver barriers are the significant inhibiting factors in i4.0 adoption, future studies should analyze how their impact can be minimized. The examination of dependent and linkage barriers in manufacturing organizations is also required. Second, the study considered only the manufacturing sector, so future researchers are encouraged to incorporate other sectors. Third, the respondents were from Pakistan, while future studies should be conducted in other countries to investigate and validate these barriers. Future studies may use Structural Equation Modeling (SEM) to validate the developed model statistically. Finally, the present study used cross-sectional data for TOPSIS, and therefore, future studies need to employ longitudinal data design in order to address the limitations of cross-sectional data.

## Supporting information

S1 Data (RAR)

S1 Appendix Table A1. Psychological barriers for Industry 4.0 [94–104].
(DOCX)
