# Supplementary material for: Developing an interplay among the psychological barriers for the adoption of industry 4.0 phenomenon
Source: PLoS One. 2021 Aug 2;16(8):e0255115. doi: 10.1371/journal.pone.0255115 (PMC8328306; doi:10.1371/journal.pone.0255115)
Supplement: S1 Appendix — Psychological barriers for Industry 4.0 [94–104]. (DOCX) [file pone.0255115.s002.docx]

**S1 Appendix**

Table A1. Psychological barriers for Industry 4.0

| **Sr. No.** | **Psychological Barriers** | **Description** |
| --- | --- | --- |
| **1** | Fear of job losses/ Employment disruptions (B1) | There is a major role of robotic and automatic technologies in adopting the i4.0 phenomenon, but this leads to employees’ replacement with these automated technologies resulting in job disruption risk [[14](#_ENREF_14), [41](#_ENREF_41)]. This is because of a dearth of understanding of the interplay between humans and technology [[94](#_ENREF_94)]. If employees are not able to satisfy the new requirements and adapt quickly enough, then there is a social risk of job losses [[43](#_ENREF_43)]. |
| **2** | Fear of outdatedness of competency (B2) | This refers to a pervasive fear among employees that after the change, their existing skills and competencies will no longer be of any use and what their role will be after the change [[45](#_ENREF_45)]. |
| **3** | Loss of face/image (B3) | There is a perception among employees that after the change, they may feel embarrassed and, thus, view change as a witness that the way they have done things in the past was erroneous [[45](#_ENREF_45)]. |
| **4** | Social risk barriers (B4) | There are perceived worries that a related social group would not approve the adoption of i4.0 [[23](#_ENREF_23), [95](#_ENREF_95), [96](#_ENREF_96)]. |
| **5** | Mismatch between industry 4.0 requirements and the institution’s capacity/ Compatibility Barrier (B5) | Factories must be redesigned to exploit i4.0 technologies [[97](#_ENREF_97)], and workers must be reallocated [[98](#_ENREF_98)]. In addition, compatibility issues may arise while upgrading the existing equipment and machines. Thus, it is considered that players with medium to small capabilities would struggle, while big players might do because the whole industry is not ready with all the technological infrastructure [[22](#_ENREF_22)]. |
| **6** | Fear of economic loss/ uncertainties about cost-effectiveness (B6) | It requires excessive initial investment to develop advanced automated technologies, but this financial initiative can play a significant role in terms of innovation, skill evolution, and job creation [[99](#_ENREF_99)]. However, there is a common psychological barrier and threat that the rapid growth of technologies would make these investments riskier [[100](#_ENREF_100), [101](#_ENREF_101)]. Due to the absence of financial resources, developing countries are prone to cost-related barriers [[17](#_ENREF_17)]. The economic benefits and productivity gains of investment in technology have always been questioned [[3](#_ENREF_3), [20](#_ENREF_20)]. Thus, there is always a significant threat of non-recovery of investments and potential financial losses for financially constrained small and medium enterprises [[22](#_ENREF_22)]. Hence, implementation cost has been reported as a critical barrier [[35](#_ENREF_35)]. |
| **7** | Conflict with their culture and values/ Norm barriers (B7) | There are norm barriers that the i4.0 conflict with family values, entrenched traditions, established opinions and social norms [[23](#_ENREF_23)]. Therefore, it becomes necessary to have a culture that is open to experimentation, and fosters innovation in order to harness the value of i4.0 [[102](#_ENREF_102)]. But the existing employees prefer status quo situations and resist any change because they are afraid of a power shift [[19](#_ENREF_19)] and feel that the person with those capabilities would be in higher demand. This deters individuals and firms from adopting i4.0 [[20](#_ENREF_20)]. During i4.0 projects, a priority task is to achieve cultural acceptance of innovations [[8](#_ENREF_8)]. |
| **8** | Lack of necessary talent (B8) | Many firms perceive and admit that they do not have the technical skills and knowledge or necessary expertise for realizing the full potential of i4.0 applications [[20](#_ENREF_20)]. According to [Geissbauer, Schrauf [103]](#_ENREF_103), the second major barrier is under-qualified employees because low-skilled employees may be less inclined to use Industry 4.0. [Horváth and Szabó [8]](#_ENREF_8) and [Breunig, Kelly [102]](#_ENREF_102) also claimed that one major challenge is a lack of talented and skilled workers with the competencies required to implement i4.0 technologies. Hence, in realizing the proper implementation of Industry 4.0, a major challenge is a lack of digital skills and an adequately skilled workforce [[14](#_ENREF_14), [17](#_ENREF_17)]. |
| **9** | Dreaded inequality (B9) | As the technology will segregate the market into high skills/high pay and low skills/low pay categories, i4.0 will bring social tension in the labor market, potentially disrupting this advancement [[46](#_ENREF_46)]. Furthermore, it is perceived that the gap between young and old generations, and between the developed and the developing nations will worsen because of i4.0 [[20](#_ENREF_20)]. |
| **10** | Realization Barrier/Uncertainty (B10) | This refers to the perceived difficulties in observing the i4.0 in use and employees being uncertain about what the future is likely to hold [[45](#_ENREF_45)] and clear comprehension motivate to adopt i4.0 [[3](#_ENREF_3)]. |
| **11** | Fear of data loss/Risk of security breaches (B11) | There are serious data security threats because the i4.0 systems are vulnerable to cyber-attack [[42](#_ENREF_42)]. In addition, there is the online availability of a large amount of confidential information across cloud computing environments. This poses a cybersecurity threat and fear to employees as other parties may assess important sensitive data. The employees must be aware of data security and cyber threat. So, this barrier needs to be addressed very prudently [[41](#_ENREF_41)]. |
| **12** | Usage barriers (B12) | Employees feel comfortable in existing routines and habits and perceive that consumption of innovation and i4.0 revolution necessitates an undesirable disruption of conventional user patterns, practices and workflows [[45](#_ENREF_45)]. Thus, usage barriers occur when an innovation or revolution is incongruent with existing habits [[47](#_ENREF_47)]. |
| **13** | Personality/Low tolerance for change (B13) | Irrespective of whether the change is beneficial and positive, some individuals have low tolerance to change due to their individual behavior and attitudes. They perceive a loss of control because the change is not by them, but it is being done to them [[45](#_ENREF_45)]. |
| **14** | No venturing motivation (B14) | If you are not motivated to bring a change, you will stop when things get complicated. Unfortunately, a common perception exists among employees that they will give up when challenges come across the implementation of i4.0 [[40](#_ENREF_40)]. |
| **15** | Lack of effective communication strategy/ Communicability barrier (B15) | A perception lies among personnel of almost all kinds of organizations that there is always an assumption of top leaders that people will adjust and be ready to get started with the new development once they announce the change. This is the silliest way because it is not only a change that employees need to know. They need to understand how they will adapt to the change and how the change will affect them regarding i4.0 benefits or shortcomings through dialogue (Joachim et al., 2018), so intensive communication is required [[8](#_ENREF_8)]. |
| **16** | Lack of a leader with appropriate skills, competencies and experience (B16) | Proper conscious planning is necessary from the very beginning of the projects. When production processes are digitized, companies need a leader with the necessary experience and skills to control i4.0 projects. There is primarily an issue of not having such a leader. Therefore, several enterprises are not yet prepared for i4.0 [[8](#_ENREF_8)]. There is also a demand for data analysts to create value through optimization and forecasting [[22](#_ENREF_22)]. |
| **17** | Lack of continued education of employees (B17) | Because of a lack of understanding of industry 4.0’s strategic importance, there is a need for continuing education of employees [[28](#_ENREF_28)]. There is also a deficiency of an advanced education system for the training of personnel [[40](#_ENREF_40)]. |
| **18** | Lack of integrated work environment among all stakeholders (B18) | There is a strong need for integration among all the channel members in industry 4.0. However, a paucity of willingness to cooperate is a major barrier but at the supply chain level. The difficulty of coordination may significantly affect the introduction of new technologies [[8](#_ENREF_8), [20](#_ENREF_20)]. |
| **19** | Lack of standards and reference architecture (B19) | A significant barrier to thriving the adoption of i4.0 is the formation of efficient architecture throughout supply chains because there is a lack of standards and reference architecture as the concept of i4.0 is new [[22](#_ENREF_22)]. In addition, an obstacle of a low degree of standardization may occur in inter-organizational relationships [[8](#_ENREF_8)]. |
| **20** | Challenges in value-chain integration (B20) | Breaking down obstacles between various organizational departments is challenging to achieve the smooth coordination needed for Industry 4.0. When integration is required among multiple organizations in the value chain, this challenge is amplified [[20](#_ENREF_20)]. Most firms fail due to poor integration in an i4.0 environment [[104](#_ENREF_104)]. Moreover, establishing interoperability and integration between different systems and technologies is very difficult to create cyber-physical infrastructure [[14](#_ENREF_14)]. |
